# Post-licensure deployment of oral cholera vaccines: a systematic review

**DOI:** 10.2471/BLT.14.139949

**Published:** 2014-09-29

**Authors:** Stephen Martin, Anna Lena Lopez, Anna Bellos, Jacqueline Deen, Mohammad Ali, Kathryn Alberti, Dang Duc Anh, Alejandro Costa, Rebecca F Grais, Dominique Legros, Francisco J Luquero, Megan B Ghai, William Perea, David A Sack

**Affiliations:** a Pandemic and Epidemic Diseases Department, World Health Organization, Geneva, Switzerland.; b University of the Philippines Manila-National Institutes of Health, Manila, Philippines.; c Delivering Oral Vaccine Effectively (DOVE), Johns Hopkins Bloomberg School of Public Health, 615 N Wolfe Street, Baltimore, MD 21205, United States of America (USA).; d United Nations Children’s Fund, United Nations Plaza, New York, USA.; e National Institute of Hygiene and Epidemiology, Hanoi, Viet Nam.; f Epicentre, Paris, France.

## Abstract

**Objective:**

To describe and analyse the characteristics of oral cholera vaccination campaigns; including location, target population, logistics, vaccine coverage and delivery costs.

**Methods:**

We searched PubMed, the World Health Organization (WHO) website and the Cochrane database with no date or language restrictions. We contacted public health personnel, experts in the field and in ministries of health and did targeted web searches.

**Findings:**

A total of 33 documents were included in the analysis. One country, Viet Nam, incorporates oral cholera vaccination into its public health programme and has administered approximately 10.9 million vaccine doses between 1997 and 2012. In addition, over 3 million doses of the two WHO pre-qualified oral cholera vaccines have been administered in more than 16 campaigns around the world between 1997 and 2014. These campaigns have either been pre-emptive or reactive and have taken place under diverse conditions, such as in refugee camps or natural disasters. Estimated two-dose coverage ranged from 46 to 88% of the target population. Approximate delivery cost per fully immunized person ranged from 0.11–3.99 United States dollars.

**Conclusion:**

Experience with oral cholera vaccination campaigns continues to increase. Public health officials may draw on this experience and conduct oral cholera vaccination campaigns more frequently.

## Résumé

**Objectif:**

Décrire et analyser les caractéristiques des campagnes de vaccination orale contre le choléra; y compris le site, la population cible, la logistique, la couverture vaccinale et les coûts de distribution.

**Méthodes:**

Nous avons effectué des recherches dans PubMed, le site Internet de l'Organisation mondiale de la Santé (OMS) et la base de données Cochrane sans aucune restriction de date ou de langue. Nous avons contacté des membres du personnel de la santé publique, des experts travaillant dans le domaine et dans les ministères de la Santé et nous avons ciblé les recherches sur Internet.

**Résultats:**

Nous avons inclus 33 documents au total dans l'analyse. Un seul pays, le Viet Nam, inclut la vaccination orale anticholérique dans son programme de santé publique et a administré environ 10,9 millions de doses de vaccins entre 1997 et 2012. En outre, plus de 3 millions de doses des deux vaccins oraux anticholériques préqualifiés de l'OMS ont été administrés dans plus de 16 campagnes de vaccination dans le monde entier entre 1997 et 2014. Ces campagnes ont été menées en prévention ou en réaction et ont eu lieu dans diverses conditions, comme dans des camps de réfugiés ou lors de catastrophes naturelles. La couverture estimée des deux doses était comprise entre 46 et 88% de la population cible. Les frais de distribution approximatifs par personne entièrement vaccinée sont compris entre 0,11 et 3,99 dollars.

**Conclusion:**

L'expérience avec les campagnes de vaccination orale contre le choléra continue à se développer. Les responsables de la santé publique peuvent tirer profit de cette expérience et mener plus fréquemment des campagnes de vaccination orale contre le choléra.

## Resumen

**Objetivo:**

Describir y analizar las características de las campañas de vacunación oral contra el cólera, incluyendo la ubicación, la población objetivo, la logística, los costes de cobertura y la entrega de vacunas.

**Métodos:**

Realizamos búsquedas en PubMed, la página web de la Organización Mundial de la salud (OMS) y la base de datos Cochrane sin restricciones de fechas ni idioma. Nos pusimos en contacto con el personal de salud pública, expertos del sector y los ministerios de salud, y realizamos búsquedas específicas en la web.

**Resultados:**

Se incluyó un total de 33 documentos en el análisis. Un país, Viet Nam, incorpora vacunas orales contra el cólera en su programa de salud pública y ha administrado aproximadamente 10,9 millones de dosis de vacunas entre 1997 y 2012. Además, se han administrado más 3 de millones de dosis de las dos vacunas orales contra el cólera orales que cumplen con los requisitos de la OMS en más de 16 campañas en todo el mundo realizadas entre 1997 y 2014. Estas campañas han sido preventivas o reactivas, y se han llevado a cabo en diversas condiciones, como en campamentos de refugiados o desastres naturales. La cobertura estimada de dos dosis osciló entre el 46 y 88 % de la población objetivo. El coste aproximado del suministro por persona completamente inmunizada osciló entre 0,11 y 3,99 dólares de los Estados Unidos.

**Conclusión:**

La experiencia con las campañas de vacunación oral contra el cólera sigue aumentando. Los funcionarios de salud pública pueden aprovechar esta experiencia y realizar campañas de vacunación orales contra el cólera con mayor frecuencia.

## ملخص

**الغرض:**

وصف خصائص حملات التطعيم بلقاحات الكوليرا الفموية وتحليلها؛ بما في ذلك الموقع والسكان المستهدفين والخدمات اللوجستية والتغطية باللقاح وتكاليف الإيتاء.

**الطريقة:**

بحثنا في PubMed وموقع منظمة الصحة العالمية على الإنترنت وقاعدة بيانات كوكرين دون قيود على التاريخ أو اللغة. واتصلنا بالعاملين في الصحة العمومية وبالخبراء في هذا المجال وفي وزارات الصحة وأجرينا بحثاً مستهدفاً على الإنترنت.

**النتائج:**

تم إدراج إجمالي 33 وثيقة في التحليل. وأدرج بلد واحد، هو فييت نام، التطعيم بلقاحات الكوليرا الفموية في برنامج الصحة العمومية وقدم 10.9 مليون جرعة لقاح تقريباً بين عامي 1997 و2012. بالإضافة إلى ذلك، تم تقديم ما يزيد عن 3 ملايين جرعة من لقاحين للكوليرا الفموية قبل اعتمادهما من منظمة الصحة العالمية في أكثر من 16 حملة في جميع أرجاء العالم بين عامي 1997 و2014. وكانت هذه الحملات إما وقائية أو تفاعلية وتم تنفيذها في ظروف شتى، مثل مخيمات اللاجئين أو الكوارث الطبيعية. وتراوح نطاق التغطية المقدرة ثنائية الجرعة من 46 إلى 88 % للسكان المستهدفين. وتراوحت تكلفة الإيتاء التقريبية لكل شخص يحصل على التطعيم الكامل من 0.11 إلى 3.99 دولاراً أمريكياً.

**الاستنتاج:**

ما زالت خبرات حملات التطعيم بلقاحات الكوليرا الفموية في تزايد. ويستطيع مسؤولو الصحة العمومية الاستناد إلى هذه الخبرات وتنفيذ حملات التطعيم بلقاحات الكوليرا الفموية بشكل أكثر تكراراً.

## 摘要

**目的:**

描述和分析口服霍乱疫苗接种活动的特点，包括位置、目标人群、物流、疫苗覆盖率和交付成本。

**方法:**

我们搜索了PubMed、世界卫生组织（WHO）网站和Cochrane数据库，搜索中不附加任何日期或语言限制。联系该领域和卫生部的公共卫生人员及专家并执行有针对性的网络搜索。

**结果:**

在分析中总共包括33个文件。越南将口服霍乱疫苗接种纳入公共卫生计划，并在1997年和2012年之间管理了大约1090万剂疫苗。此外，在1997年和2014年之间，世界各地超过16个运动中已管理超过300万剂量的两个WHO资格预审的口服霍乱疫苗。这些运动有主动出击式，也有被动反应式，并在不同条件下实施，比如在难民营或发生自然灾害时。估计目标人群中两剂量的覆盖范围为46%到88%。完全免疫的个人近似交付成本范围为0.11-3.99美元。

**结论:**

口服霍乱疫苗接种活动的经验持续增加。公共卫生官员可以利用这种经验，更经常性地进行口服霍乱疫苗接种活动。

## Резюме

**Цель:**

Описать и проанализировать особенности кампаний по проведению пероральной вакцинации против холеры, включая места проведения, целевые группы населения, логистику, охват вакцинацией и стоимость доставки вакцины.

**Методы:**

Поиск был осуществлен в базах данных PubMed, на сайте Всемирной организации здравоохранения (ВОЗ) и в Кокрановской базе данных без каких-либо ограничений по датам или языку. Были проведены беседы с сотрудниками органов здравоохранения, экспертами в данной области и в министерствах здравоохранения, а также проведен целенаправленный поиск через поисковые системы в Интернете.

**Результаты:**

Всего было проанализировано 33 документа. В одной стране, во Вьетнаме, пероральная вакцинация против холеры является частью программы здравоохранения, и в период с 1997 по 2012 гг. было введено приблизительно 10,9 миллиона доз вакцины. Кроме того, более 3 миллионов доз двух пероральных противохолерных вакцин, прошедших предварительную оценку ВОЗ на соответствие требованиям, были введены в ходе более 16 кампаний по всему миру в период с 1997 по 2014 гг. Эти кампании носили либо превентивный, либо реактивный характер и проводились в разных условиях, например в лагерях беженцев или на месте природных катастроф. По приблизительным подсчетам по две дозы получили 46-88% целевой группы населения. Приблизительная стоимость доставки в расчете на одного прошедшего полную иммунизацию человека варьировалась в пределах от 0,11 до 3,99 долларов США.

**Вывод:**

Опыт проведения кампаний по пероральной вакцинации против холеры продолжает накапливаться. Официальные лица органов здравоохранения могут использовать данный опыт и чаще проводить кампании по пероральной вакцинации против холеры.

## Introduction

*Vibrio cholerae* O1 and O139 causes severe diarrhoea and the main strategies to prevent the disease are to promote hygiene and to ensure safe water and sanitation. These basic needs are often not met in endemic areas with seasonal cholera outbreaks or during man-made or natural disasters in impoverished areas. An additional tool for cholera prevention and control is the oral cholera vaccine. In October 2009, the World Health Organization (WHO) Strategic Advisory Group of Experts on immunization recommended that oral cholera vaccination should be considered as a reactive strategy during outbreaks, in addition to the already recommended preventive use of oral cholera vaccine in endemic areas.^1^ A vaccine stockpile was created in 2012, with an initial two million doses to be available mainly for epidemic response in low-income countries.^2^ In November 2013, the global alliance for vaccines and immunizations (Gavi Alliance) approved a financial contribution towards the stockpile to expand its use. With the availability of the oral cholera vaccine stockpile, more governments might consider cholera vaccination where needed.

A monovalent inactivated vaccine containing killed whole-cells of *V. cholerae* serogroup O1 and the B-subunit of cholera toxin was the first oral cholera vaccine to obtain international licensure in 1991 and WHO prequalification in 2001. The vaccine is marketed as Dukoral® (Crucell, Netherlands). Randomized, placebo-controlled trials of earlier versions of Dukoral® in Bangladesh and the current recombinant B-subunit whole cell vaccine in Peru showed that the vaccine is safe and confers an initial protection of approximately 85% in the first months.^3^^,^^4^ Follow-up studies in Bangladesh estimated a 62% protection during the first year, 57% during the second year and negligible thereafter.^3^

During the mid-1980s, the National Institute of Hygiene and Epidemiology in Viet Nam developed an oral cholera vaccine for the country’s public health programme. A two-dose regimen of a first-generation of monovalent (anti-O1) cholera vaccine had an estimated efficacy of 66% against the El Tor strain of *V. cholerae*.^5^ In 1997, the vaccine was augmented with killed *V. cholerae* serogroup O139 whole cells to create a bivalent vaccine,^6^ which was locally licensed as ORC-Vax™ (Vabiotech, Viet Nam). After changing production procedures in 2009, the vaccine was reformulated and licensed as mORC-Vax™ (Vabiotech, Viet Nam) and is currently used in Viet Nam’s public health programme.^7^ However, the vaccine is not pre-qualified by WHO.

To make the mORC-Vax™ internationally available, manufacture of the reformulated vaccine was transferred to Shantha Biotechnics Ltd in India, where the national regulatory authority is approved by WHO.^8^ This led to the development of Shanchol™, which is the third currently-available oral cholera vaccine. A randomized, placebo-controlled trial in India showed that Shanchol™ is safe and confers 67% protective efficacy against cholera within two years of vaccination,^8^ 66% at three years^9^ and 65% at five years^10^ of follow-up. Shanchol™ was licensed in India in 2009 and received WHO pre-qualification in 2011.

A comparison of the three oral cholera vaccines is shown in Table 1.^11^^,^^12^ The safety, relative effectiveness and duration of protection of the different types of oral cholera vaccine has previously been reviewed.^13^ Here we conduct a systematic review of post-licensure oral cholera vaccines. The objective of the review is to generate information – by describing and analysing the campaigns – that can be used to inform planning for the future use of these vaccines.

**Table 1:** Oral cholera vaccines, 2014

Vaccine	Dukoral®^11^	ORC-Vax™ and mORC-Vax™^11^^,^^12^	Shanchol™^11^
Manufacturer	Crucell (the Netherlands)	Vabiotech (Viet Nam)	Shantha Biotechnics Ltd (India)
Description	Monovalent inactivated vaccine	Bivalent inactivated vaccine	Bivalent inactivated vaccine
Components	Killed whole-cells of *V. cholerae* O1 (Classical and El Tor biotypes) and recombinant B-subunit of cholera toxin	Killed whole cells of *V. cholerae* O1 (Classical and El Tor biotypes) and *V. cholerae* O139	Killed whole cells of *V. cholerae* O1 (Classical and El Tor biotypes) and *V. cholerae* O139
Recommended age	2 years and older	1 year and older	1 year and older
Delivery	Oral	Oral	Oral
Doses	Two doses ≥ 1 week apart	Two doses ≥ 2 weeks apart	Two doses ≥ 2 weeks apart
Buffer	Yes. Buffer dissolved in 75 mL (2–6 years old) or 150 mL (> 6 years old) water	Not required	Not required
Licensure	International (1991)	Viet Nam (1997/2009)	India (2009)
WHO pre-qualification	Yes (2001)	No	Yes (2011)
Storage temperature	2–8 °C	2–8 °C	2–8 °C

## Methods

### Search

We searched the Cochrane database of systematic reviews and its database of abstracts and reviews of effects from 1990 to the present and found no reviews of oral cholera vaccination campaigns.

We conducted a systematic review of published documents on post-licensure vaccination campaigns using one of three oral cholera vaccines following the search and analysis process recommended in the Preferred Reporting Items for Systematic Reviews and Meta-analyses guidelines. We searched PubMed and the WHO website using “cholera vaccination”, “cholera outbreak response” and “cholera vaccination campaign” as search terms with no date or language restrictions. The bibliographies of the retrieved articles were also screened for relevant papers. Reports, presentations and international organization or company documents were obtained through targeted web searches. We also contacted public health personnel, experts in the field and in ministries of health for further information.

All identified documents in English that described campaigns using oral cholera vaccine were assessed for appropriateness using the following selection criteria. We included all documents describing campaigns using Dukoral® after 1991, ORC-Vax™ after 1997, mORC-Vax™ after 2009 and Shanchol™ after 2009. Campaigns organized either as part of a public health response to endemic or epidemic cholera, pilot campaigns, demonstration projects, assessments of feasibility and acceptability, as well as studies of vaccine effectiveness were included. Each campaign may have more than one reference, describing different aspects of the vaccination (e.g. feasibility, coverage, cost, etc.). We excluded documents describing pre-licensure trials, reports on knowledge and perception of cholera and oral cholera vaccines, as well as planning or policy briefs that did not describe actual oral cholera vaccine deployment.

By adhering to the pre-defined inclusion and exclusion criteria, we could make a valid comparison across articles. To assess the broad picture of the vaccine campaigns, we did not exclude any document based on quality or deficiency of reporting. Information from the published and unpublished documents was extracted and entered into a spreadsheet independently by two of the authors and then corroborated and summarized by a third author.

### Definitions

Oral cholera vaccine campaigns can either be pre-emptive or reactive. Pre-emptive or preventive vaccination refers to campaign implementation before a cholera outbreak begins, ideally in conjunction with improved water, hygiene and sanitation. Pre-emptive vaccination may be conducted before the next seasonal outbreak in sites where cholera regularly occurs, in communities adjacent to an area with cholera or during humanitarian emergencies to prevent cholera. Reactive campaigns are those implemented after a cholera outbreak has started and while cholera cases are still being detected in the target population.^14^ In areas where cholera tends to occur all year-round, the distinction between pre-emptive and reactive vaccination may be difficult.

The target population was defined as the number of individuals living in a circumscribed area to whom oral cholera vaccine is offered. The target population may be an estimate based on administrative population figures or a more precise figure based on a study census. Coverage was defined as the percentage of the target population who received one dose and two doses (fully immunized) of the vaccine, except when otherwise indicated (i.e. community surveys were used to calculate vaccine coverage in some campaigns particularly when a precise target population number was not known). The approximate total number of oral cholera vaccine doses deployed was defined as the sum of the first and second dose recipients; when data on the first dose recipients were not available, we multiplied the number of fully vaccinated individuals by two. We plotted the number of approximate doses deployed in oral cholera vaccine campaigns by country. Countries were colour-coded by the number of cholera cases reported in 2005,^15^ using ArcMap 10.0 (ESRI, Redlands, USA). Adverse events following immunization were defined as medical incidents that take place after an immunization and cause concern. Adverse events following immunization may be coincidental or causally associated. A serious adverse event following immunization is one that requires hospitalization and/or causes birth defects, permanent damage, or death.

To allow comparison of the expenses for vaccination across various campaigns, the expenses were grouped into the following categories: vaccine and/or international shipment costs, computers and other capital expenses, international consultants, local storage and transport, meetings, social mobilization, training, local salaries, supplies and waste management and the detection and management of adverse events following immunization. The delivery cost per fully immunized person was calculated using the total local expenses (excluding vaccine, international shipment and consultant costs) as the numerator and the number of fully immunized persons as the denominator.

## Results

**Fig. 1:**
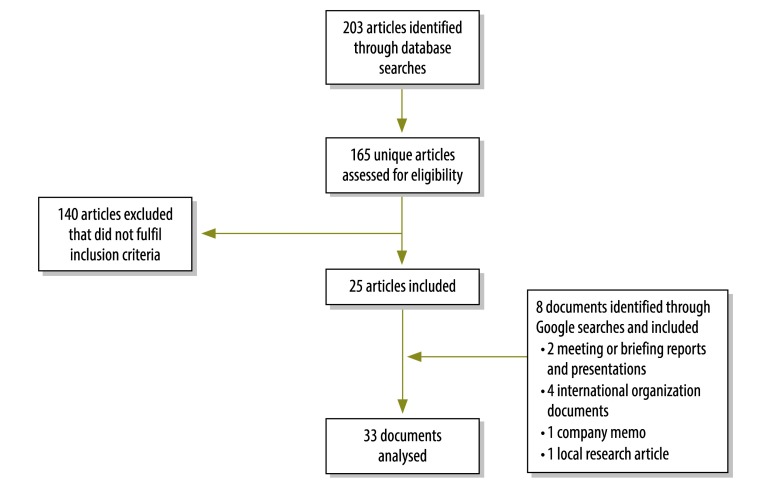
Flowchart for the selection of documents on oral cholera vaccination campaigns

**Fig. 2:**
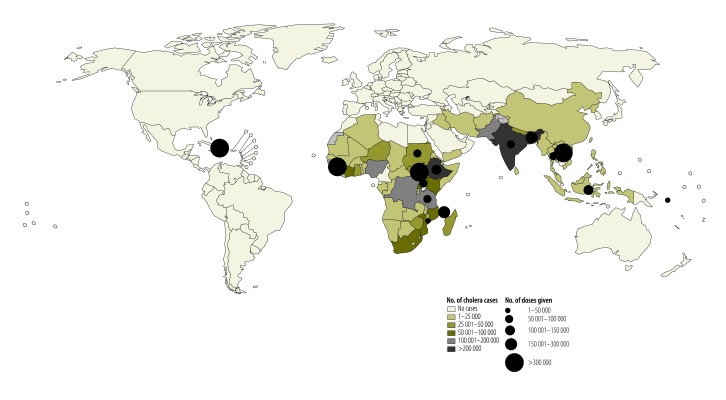
Post-licensure oral cholera vaccination campaigns, 1997–2014

**Fig. 3:**
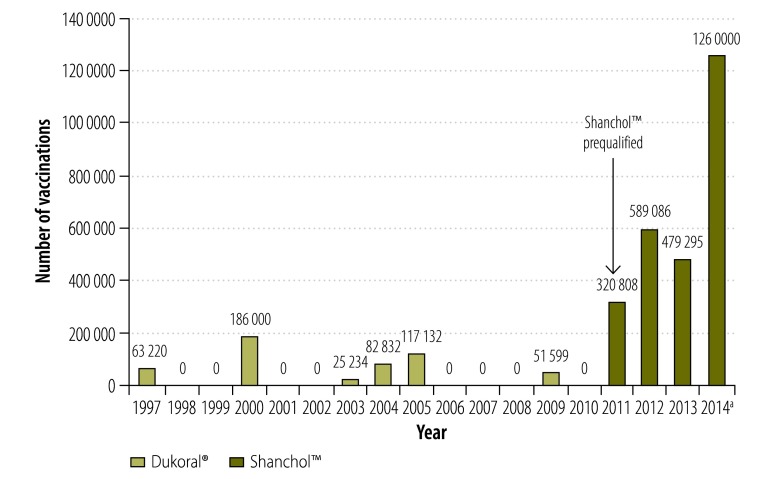
Administration of Dukoral® or Shanchol™ in post-licensure oral cholera vaccination campaigns globally, 1997–2014

^a^ Number of vaccinations in 2014 counted from January to August.

We identified 173 unique documents of potential relevance and 33 of these met the inclusion criteria (Fig. 1).^16^^–^^48^ In addition, we obtained information about recent campaigns through personal communications with two co-authors (DL and KA). We mapped the approximate number of doses administered in post-licensure oral cholera vaccination campaigns from 1997 to 2014 (Fig. 2) and plotted them by year (Fig. 3). As of August 2014, 280 000 oral cholera vaccine doses from the stockpile were shipped to Ethiopia, 280 000 to Guinea, 400 000 to Haiti and 300 000 to South Sudan. For campaigns with detailed data available, the characteristics and main findings are shown in Table 2 and the vaccination logistics by target population size is shown in Table 3.

**Table 2:** Characteristics and main findings of post-licensure oral cholera vaccination campaign studies, 1997–2014

Vaccine and year of the campaign	Site	Setting	Type and purpose of the vaccination campaign	Eligibility criteria	Target population	Coverage			Main findings
						Received 1st dose, no. (%)	Received 2nd dose, no. (%)		
**Dukoral®**									
1997	Adjumani district, Uganda	Refugee camp, rural	Pre-emptive vaccination to assess feasibility in a stable refugee camp setting^16^^,^^17^	≥ 1 year old	44 000	35 613 (81)	27 607 (62)		Oral cholera vaccination of a large refugee population is feasible.^16^ During a cholera epidemic in the area the following year, cholera attack rates were 0.59% in the non-refugee Ugandan villages, 0.04% in the 30 non-vaccinated refugee camps and 0.00% in the six vaccinated refugee camps^17^
2000	Mayotte Island, Comoros	Urban and rural	Pre-emptive vaccination campaign to prevent a cholera epidemic^18^	NA	145 000	NA	93 000 (64)		NA
2003–2004	Beira, Mozambique	Urban	Pre-emptive vaccination in an endemic area with seasonal outbreaks. Effectiveness study in an HIV-endemic sub-Saharan African site^20^^,^^21^	Non-pregnant women, ≥ 2 years old children	19 550	14 164 (72)	11 070 (57)		Mass vaccination was feasible but required considerable logistic support and planning.^20^ One or more doses conferred 78% protection (95% CI: 39–92) against cholera during the year post vaccination^21^
2004	South Darfur, Sudan	Refugee camp, rural	Pre-emptive vaccination to assess feasibility during the acute phase of an emergency (i.e. refugee camp of internally displaced persons)^22^^,^^23^	≥ 2 years old	45 825	42 502 (93)	40 330 (88)		Although planning and implementation requirements were significant, the campaign was successful because of the strong support and commitment of the refugee community and collaborators^22^^,^^23^
2005	Aceh, Indonesia	Site of internally displaced persons	Pre-emptive vaccination to assess feasibility during the acute phase of an emergency (i.e. post-tsunami)^23^^,^^24^	≥ 2 years old	78 870	62 505 (79)	54 627 (69)		Challenges in the coordination, heavy logistics and frequent aftershocks complicated and delayed implementation. Difficulties in maintaining a cold chain resulted in 11.7% vaccine losses^23^^,^^24^
2009	Zanzibar, the United Republic of Tanzania	Urban and rural	Pre-emptive vaccination in an endemic area with seasonal outbreaks. Effectiveness study to measure direct and indirect protection^26^^–^^28^	Non-pregnant women, ≥ 2 years old children	48 178	27 678 (57)	23 921 (50)		Confirmed direct vaccine effectiveness of 79% (95% CI: 47–92). First study to show vaccine herd protection in an African setting: 75% (95% CI: 11–93%) indirect protection in the higher coverage group compared with the lower coverage group.^26^ No evidence of a harmful effect of gestational exposure to the vaccine.^27^ First use of personal digital assistants for direct data entry during a survey enumeration and mass vaccination^28^
**ORC-Vax**™** and mORC-Vax**™									
1998–2012	Viet Nam	Endemic urban and rural areas	Pre-emptive and reactive vaccinations of children integrated into the country’s public health programme^33^	Non-pregnant women, ≥ 1 year old children	≈10.9 million doses	NA	NA		Viet Nam is the only country in the world to regularly use oral cholera vaccinations. Since 1997, the number of cholera cases in Viet Nam has declined, in association with increased vaccination use as well as improvements in socioeconomic and water and sanitation conditions^33^
1998 and 2000	Hue, Viet Nam	Urban and rural	Pre-emptive vaccination campaign in a cholera-endemic area. Study to assess long term effectiveness^30^^,^^31^	Non-pregnant women, ≥ 1 year old children	149 557 (1998) and 137 082 (2000)	In 1998: 125 135 (84) and in 2000:104 706 (76)	In 1998:118 703 (79) and in 2000:103 226 (75)		Mass immunization is feasibly administered through the public health system.^30^ Direct vaccine effectiveness 3 to 5 years after vaccination was 50% (95% CI: 9–63)^31^
2008	Hanoi, Viet Nam	Urban	Reactive vaccination campaign during an on-going outbreak^32^	Non-pregnant women, ≥ 1 year old children	≈370 000 > 10 years old	NA	≈80% vaccinated		Protective effectiveness of 76% (95% CI: 5–94). First study to document reactive use of oral cholera vaccination during an outbreak^32^
**Shanchol**™									
2011	Odisha, India	Rural	Pre-emptive vaccination campaign and feasibility study^34^	Non-pregnant woman, ≥ 1 year old	51 488	31 552 (61)	23 751 (46)		Feasible to vaccinate using governmental set-up^34^
2011	Dhaka, Bangladesh	Endemic urban areas	Pre-emptive vaccination. Cluster randomized study with three arms: vaccine, vaccine plus safe water and hand washing practice and no intervention^35^	Non-pregnant women, ≥ 1 year old children	172 754	141 839 (82)	123 666 (72)		Feasible to use the national immunization set-up.^35^ On-going study of vaccine effectiveness
2012	Port-au-Prince, Haiti	Urban	Reactive vaccination campaign. Pilot study^36^	≥ 1 year old children	70 000	52 357 (75)	47 540 (68)		Effort, community mobilization and organizational capacity needed for a successful campaign where there were logistical and security challenges^36^
2012	Bocozel and Grand Saline, Haiti	Rural	Reactive vaccination campaign. Pilot study^37^^–^^40^	≥ 1 year old children	≈50 000	45 417	41 238 (Estimated 77–79% in Bocozel and 63% in Grand Saline)		The campaign integrated with the other components of cholera control was found to be feasible and acceptable^37^^–^^40^
2012	Choiseul and Shortland, Solomon Islands	Rural	Pre-emptive vaccination campaign near an area with a cholera outbreak^41^	Children 1–14 years old in high-risk areas	NA	11 888	11 318		NA
2012	Tak Province, Thailand	Refugee camps, rural	Pre-emptive vaccination campaign with a knowledge, attitudes and practices survey^42^	Non-pregnant women, ≥ 1 year old children	43 968	36 325 (83)	26 753 (61)		First use of Shanchol™ in a stable refugee camp setting^42^
2012	Boffa and Forecariah regions, Guinea	Rural	Reactive vaccination campaign during an on-going outbreak and feasibility study^43^^–^^45^	≥ 1 year old children	≈209 000 (≈163 000 in Boffa and ≈46 000 Forecariah)	172 544	143 706 (Based on administrative population figures, 68% in Boffa and 51% in Forecariah. Household survey immediately after campaign 76%)^43^		First use of Shanchol™ in sub-Saharan Africa. The campaign was successful despite short preparation time, remote rural setting and highly mobile population.^43^^,^^44^ Protective effectiveness of 87% (95% CI: 56–96)^45^
2013	Maban county, South Sudan	Refugee camps, rural	Pre-emptive vaccination campaign in an area with escalating Hep E outbreak^46^^,^^47^	≥ 1 year old children	146 317	NA	132 000 (> 85% by survey)		The campaign was successful despite logistical challenges^46^^,^^47^
2013	Petite Anse and Cerca Carvajal, Haiti	Urban and rural	Pre-emptive vaccination campaign in a cholera-endemic area^a^	≥ 1 year old children	> 110 000	113 045	102 250		NA
2014	South Sudan	Internally displaced persons camps	Pre-emptive vaccination campaign^48^	Non pregnant women, ≥ 1 year old children	152 000	125 311 (72)	76 088 (awaiting coverage surveys)		Humanitarian crisis. First use of global OCV stockpile. Fixed and mobile teams. Second round in one site was co-administered with meningitis vaccine^48^

CI: confidence interval; Hep E: Hepatitis E; NA: information not available; OCV: oral cholera vaccination.

^a^ Information obtained through personal communications with Kathryn Alberti, UNICEF, New York, USA.

**Table 3:** Logistics of oral cholera vaccination campaigns, 1997–2013

Target population size	Site, year	Vaccine	Max. days per round	Total duration	Delivery method	Approximate doses delivered/day	Staff
< 50 000	Adjumani district, Uganda, 1997^16^	Dukoral®	4	Just over 1 month	15 vaccination sites	250–1735	114 persons: 19 nurses/midwives, 21 nursing aides, 44 community health workers and 30 persons without qualifications
	Esturro, Beira, Mozambique, 2003–2004^20^	Dukoral®	9	1 month	Outposts in churches and schools 08:00–15:00 6 days/week	Average 609	One supervisor and 15–23 members per outpost
	Zanzibar, the United Republic of Tanzania, 2009^26^	Dukoral®	15	Just over 1 month	Eight vaccination posts on each of the two islands. 8 hours daily	NA	Local health care workers and villagers
	Aceh, Indonesia, 2005^23^^,^^24^	Dukoral®	NA	5 months	Three-phase approach, three different geographical areas with approximately one month between each phase. Fixed vaccination sites with some door-to-door mop-up	100–250	4 members per team
50 000 to 100 000	Odisha, India, 2011^34^	Shanchol™	3	1 month	Vaccination booths within 10–15 minute walking distance from villagers open 07:00–17:00 daily	NA	At each booth: 1 midwife and 5–6 community health workers/volunteers
	City of God, Port-au-Prince and Bocozel and Grand Saline, Artibonite Department, Haiti, 2012^36^^,^^38^	Shanchol™	Urban: NA Rural: 10	3 months per site	Urban: door-to-door pre-registration and vaccination at 9 fixed sites. Rural: fixed posts, mobile posts and door-to-door	NA	Urban campaign: 500 staff, 75 teams of 4 workers, plus 15 supervisors Rural: 40 teams of 4 workers each led by 20 supervisors
	Viet Nam 1998 and 2000^30^^,^^31^	ORC-Vax™	9	1 month	Specifically designated sites, also used by EPI. 90 sites	139 (max)	90 teams
> 100 000	Viet Nam 2008^32^	ORC-Vax™	3	13 days	Commune health centres	NA	NA
	Mirpur, Dhaka, Bangladesh 2011^35^	Shanchol™	3-day cycles	One and half months	Fixed outreach vaccination sites. Sixty vaccine clusters were grouped into five cycles. In each 3-day vaccination cycle, 12 clusters were covered. The teams then moved on to the next cycle and thus all clusters were covered two times in two rounds	900–1000	76 vaccinators, 220 volunteers and 12 first line supervisors
	Boffa and Forecariah regions, Guinea 2012^43^^,^^44^	Shanchol™	6	3 months	Decentralized semi-mobile strategy. Most sites in place for only 1 day. In rural areas, teams could cover three sites in one day	774 (avg)	43 teams of 9 to 20 people
	Maban county, South Sudan 2013^46^^,^^47^	Shanchol™	7	Just over 1 month	Semi-mobile strategy, fixed points for first days of round, then mix of fixed sites and mop-up for last days of round. Also, in each MSF clinic	1150	Teams of 10 people at each site, plus 14 people per camp for mobilization

EPI: Expanded Programme on Immunization; MSF: Médecins Sans FrontièresNA. OCV: oral cholera vaccine.

### Dukoral®

About 526 017 doses of Dukoral® were administered in six vaccination campaigns from 1997 to 2009, all of which were pre-emptive (Table 2).^16^^–^^29^ These included two feasibility studies in refugee camps^16^^,^^17^^,^^22^^,^^23^ and one campaign following a natural disaster.^23^^,^^24^ The percentage of fully immunized persons ranged from 50–88%. There were two effectiveness studies in sub-Saharan Africa, which confirmed direct vaccine protection of 78–79%, 12 to 15 months following vaccination,^21^^,^^26^ as well as herd protection.^26^ We found one document stating that 137 000 Dukoral® doses were delivered to Myanmar in 2008^18^ but we were unable to find more information.

The duration of the vaccination campaigns ranged from one to five months and consisted of two rounds at a 10- to 14-day interval (Table 3). Each round took 4 to 15 days.^16^^,^^20^^,^^23^^,^^24^^,^^26^ A cold chain for vaccine delivery was reportedly maintained at 2–8 **°**C from storage to administration in Aceh, Indonesia,^24^ Beira, Mozambique^20^ and Zanzibar, United Republic of Tanzania.^26^ In Uganda, the vaccine was maintained at room temperature.^16^ Vaccination teams were able to vaccinate 100 to 1735 persons per day.^16^^,^^20^^,^^23^^,^^24^^,^^26^ Reported adverse events following immunization in Mozambique^20^ and Uganda^16^ were minor and non-specific. Delivery cost per fully immunized person ranged from 0.53 United States dollars (US$) to US$ 3.66 (Table 4).

**Table 4:** Cost of post-licensure oral cholera vaccinations, 1997–2013

Characteristic	Uganda, 1997^16^	Mozambique,^a^ 2003–2004^20^	Indonesia, 2005^23^^,^^24^	United Republic of Tanzania, 2009^29^	India,^a^ 2011^34^	Bangladesh, 2011^35^	Guinea, 2012^44^	South Sudan, 2013^46^
Oral cholera vaccine	Dukoral®	Dukoral®	Dukoral®	Dukoral®	Shanchol™	Shanchol™	Shanchol™	Shanchol™
Price per vaccine dose, US$	Free	Free	4.70	5.00	2.22	1.00	1.85^b^	2.40^b^
Number fully immunized persons	27 607	44 156	54 627	23 921	23 751	123 666	143 706	71 912
Vaccine and/or international shipment costs, US$	4 421	6 608	665 247	555 000	122 629	284 529	632 782^b^	661 690^b^
Computers and other capital expenses, US$	1 600	900	4 738	NA	NA	NA	NA	NA
International consultants, US$	NA	NA	124 230	110 000	NA	NA	NA	133 917^b^
Local storage and transport, US$	3 239	33 510	5 159	NA	2 081	43 701	175 930^b^	115 428^b^
Meetings, community mobilization, training, local salaries, supplies and waste management, US$	5 395	54 269	159 275	87 500	20 625^c^	157 932	106 630^b^	171 766^b^
Adverse event following immunization monitoring and management, US$	NA	NA	NA	NA	4 237	NA	NA	NA
Total cost for the vaccination campaign, US$	14 655 (0.53)	95 287 (2.16)	958 649 (17.55)	752 500 (31.46)	149 572 (6.30)	486 162 (3.93)	915 342 (6.37)^b^	1 082 801 (15.06)^b^
Total local delivery cost (per person), US$^d^	14 655 (0.53)	88 679 (2.01)	169 172 (3.10)	87 500 (3.66)	26 943 (1.13)	201 633 (1.63)	282 560 (1.97)^b^	287 197 (3.99)^b^

NA: not available; US$: United States dollar.

^a^ Including vaccinations outside the study target population.

^b^ Costs originally reported in Euro. US$ was calculated using the conversion rate as of 1 February 2013: 1 Euro to US$ 1.37.

^c^ Itemized as follows: Social mobilization US$ 5603 and vaccine administration US$ 15 022.

^d^ Excluding vaccine, international shipment and consultant costs.

### ORC-Vax™ and mORC-Vax™

In Viet Nam, an estimated 10.9 million doses of ORC-Vax™ and mORC-VAX™ have been deployed from 1997 to 2013 through targeted mass vaccination or – to children – through the Expanded Programme of Immunization in cholera-endemic regions.^30^^–^^33^ Documented coverage during the vaccination of half of the communes in Hue was 79% (118 703/149 557) in 1998 and 75% (103 226/137 082) in the other half in 2000; long term vaccine effectiveness (three to five years after the campaign) was 50%.^30^^,^^31^ (Table 2).Vaccine coverage was not precisely quantified in the 2008 Hanoi campaign; vaccine effectiveness was 76%.^32^ The duration of the vaccination campaigns ranged from two to four weeks with each round taking 3 to 9 days (Table 3).^30^^–^^32^ Mass campaigns are held yearly in Hue and are part of the routine public health provision, requiring minimal additional costs. The delivery cost in Hue during a 2013 campaign was US$ 0.11 per fully immunized person.^33^

### Shanchol™

Since WHO pre-qualification, Shanchol™ has been increasingly used in campaigns.^34^^–^^48^ About 2 649 189 doses have been administered in more than 10 campaigns (Table 2; data from the most recent campaigns in Ethiopia, Guinea and Haiti are not yet available), three of which were described as reactive. The percentage of fully immunized persons ranged from approximately 46–85% (Table 2). A study in Odisha, India 2011, found that oral cholera vaccination through the Indian public health system is feasible.^34^ The campaign in Dhaka, Bangladesh 2011, includes an assessment of vaccine effectiveness with and without other interventions.^35^ The two vaccination campaigns in Haiti in 2012 were pilot projects that paved the way for the launching of a national cholera vaccination programme integrated in a long-term plan to address water safety and sanitation.^36^^–^^40^ There was a third campaign in Haiti in 2013 that was part of this plan. Shanchol™ was deployed for pre-emptive vaccination in the Solomon Islands in 2012, following reports of cholera in a nearby area.^41^ The vaccination campaign in Thailand, 2012, was conducted to prevent seasonal outbreaks in a stable camp setting.^42^ The vaccination campaign in Guinea, 2012, was the first reactive oral cholera vaccine campaign in sub-Saharan Africa and the first time that Shanchol™ was used in an African setting.^43^^–^^45^ The campaigns in Guinea and in Maban county, South Sudan 2013 confirmed that large-scale vaccinations under logistically difficult conditions are feasible.^46^^,^^47^ The campaign in internally displaced persons camps in South Sudan in 2014, was the first to use the oral cholera vaccine stockpile.^48^

The Shanchol™ campaigns were conducted in 1–3 months.^34^^–^^48^ The 2012 Haiti campaign was carried out in two phases due to an overlapping national oral polio vaccination campaign.^36^^–^^40^ The number of persons vaccinated per day ranged from 774–1150.^35^^,^^43^^–^^48^ No serious adverse events following immunization were reported. In campaigns in Odisha, Dhaka and in Haiti in 2012, acold chain for vaccine was maintained at 2–8 **°**C from storage to delivery on site.^34^^–^^40^ In the campaigns in Guinea and in 2013 in South Sudan cold chain was maintained until the day of vaccination, during which vaccines were transported to vaccination sites and used at ambient temperature^43^^–^^47^ (Table 3).

The delivery costs of Shanchol™ through the existing government health system in Bangladesh^35^ and India^34^ were US$ 1.63 and US$ 1.13, respectively, per fully immunized person. The local expenses of reactive deployment in Guinea were US$ 1.97,^45^ while costs in Maban, South Sudan were US$ 3.99 per fully immunized person (Table 4).^47^

## Discussion

We estimate that about 3 175 206 doses of Dukoral® and Shanchol™ have been deployed in vaccination campaigns in areas affected by cholera around the world from 1997 to 2014. Only one country, Viet Nam, incorporates oral cholera vaccination into its public health programme and has used more than 10 million doses since 1997. Recently larger numbers of doses have been deployed in different areas globally but the vaccine is still under-used compared to the 1.4 billion people at risk of cholera in endemic areas.^15^ There is a shortage of licensed, WHO-prequalified cholera vaccines to meet global endemic and epidemic needs and insufficient supply is often cited as an obstacle to wider vaccine use.^49^ Availability of an oral cholera vaccine stockpile may lead to a larger vaccine supply through more consistent and predictable demands and may help increase vaccine use. Insufficient vaccine supply can be addressed by encouraging manufacturers to increase production capacity.

The deployments of oral cholera vaccine have previously been pre-emptive but recent experiences in Guinea^43^^–^^45^ and Haiti^36^^–^^40^ have shown that reactive mass vaccinations are feasible.^,^ The number of cases and deaths that can be prevented by reactive vaccination depends on the characteristics of the outbreak, with greatest impact during large and long-lasting outbreaks usually seen in populations with no recent exposure to the disease.^14^ With the development of an oral cholera vaccine stockpile and possibility of rapid deployment, increased reactive use of oral cholera vaccine is anticipated.

To be able to compare the campaigns, we calculated the total delivery cost per fully immunized person by excluding the expenditures for vaccine, shipment and technical experts, but the estimates still varied considerably. Deployment costs were lowest in Hue, Viet Nam, where the vaccine is administered routinely through the public health system^30^^,^^33^ but a similar delivery strategy may not be possible in other cholera-endemic areas or during the acute phase of emergencies. The requirement for co-administration of a buffer with the Dukoral® vaccine complicates the delivery of such vaccine and likely increases its delivery costs. Both mORC-Vax™ and Shanchol™ do not require a buffer, which should streamline the delivery and reduce logistical requirements.

This analysis has several limitations. First, there was a wide variation in the methods used to calculate coverage and costs in the vaccination campaigns. Some coverage estimations were precise, while others were approximations. Although we attempted to make the costing comparable, the calculated figures should be interpreted with caution. There are large variations in the costing of some items that cannot merely be explained by differences in site conditions and access. There are also local variables such as distance from central storage to the vaccine administration sites, campaign duration and vaccine storage conditions that affect the costs. Variations in campaign logistics also influence the estimates. Differences may also arise from the methods used to calculate expenses. For future campaigns, estimating cost using a standardized method would be very useful. Second, reporting was not consistent, as some information about the campaign, such as coverage, delivery, adverse events following immunization monitoring and other details, were not always measured or reported. We obtained the least information on the oral cholera vaccine campaigns in the Comoros and the Solomon Islands. Third, information from the more recent post-licensure vaccination campaigns is not yet available. Updated reporting will be required. Fourth, 24% (8/33) of documents included in the analysis were not published in peer-reviewed journals but were the only available sources of data for some of the vaccination campaigns. Fifth, many of the campaigns were done in collaboration between ministries of health and external health agencies (e.g. Médecins Sans Frontières, WHO, Partners for Health, United States’ Centers for Disease Control and Prevention). It will be important to continue to monitor and evaluate future campaigns using vaccine from the stockpile and implemented mainly by ministries of health.

Despite these limitations, our findings provide important lessons. The number of oral cholera vaccination campaigns is increasing and experience has been documented in a variety of settings. The increasing use of oral cholera vaccine is reassuring but more needs to be done to encourage its use where needed. Since the creation of the stockpile, a higher number of doses have been used and this increase will likely continue with the availability of an oral cholera vaccine stockpile and as more experience is gained with campaigns. Data from the deployments confirm the effectiveness, safety and feasibility of mass oral cholera vaccination. While the two-dose vaccination schedule may be perceived as an impediment to delivery and coverage, the experience with both Dukoral® and Shanchol™ disproves this perception. In addition, community education on cholera control and distribution of other preventive measures such as soap and chlorine solution were feasibly integrated into recent vaccination campaigns.^35^^,^^37^^–^^39^^,^^43^^–^^45^ We also found that there were substantial differences in how the campaigns were reported making comparisons difficult. A more systematic approach to decision-making – such as a rapid assessment tool – and a standardized method for data collection, monitoring and evaluation should be pursued, supported and published. This will ensure appropriate documentation of future campaigns.
